# Beneficial Effects of Phenolic Compounds on Gut Microbiota and Metabolic Syndrome

**DOI:** 10.3390/ijms22073715

**Published:** 2021-04-02

**Authors:** Kamila Kasprzak-Drozd, Tomasz Oniszczuk, Mateusz Stasiak, Anna Oniszczuk

**Affiliations:** 1 Department of Inorganic Chemistry, Medical University of Lublin, Chodźki 4a, 20-093 Lublin, Poland; 2 Department of Thermal Technology and Food Process Engineering, University of Life Sciences in Lublin, Głęboka 31, 20-612 Lublin, Poland; 3 Institute of Agrophysics, Polish Academy of Sciences, Doświadczalna 4, 20-290 Lublin, Poland; m.stasiak@ipan.lublin.pl

**Keywords:** gut microbiota, dysbiosis, polyphenols, metabolic syndrome, metabolic diseases, type 2 diabetes, obesity, cardiovascular diseases

## Abstract

The human intestine contains an intricate community of microorganisms, referred to as the gut microbiota (GM), which plays a pivotal role in host homeostasis. Multiple factors could interfere with this delicate balance, including genetics, age, medicines and environmental factors, particularly diet. Growing evidence supports the involvement of GM dysbiosis in gastrointestinal (GI) and extraintestinal metabolic diseases. The beneficial effects of dietary polyphenols in preventing metabolic diseases have been subjected to intense investigation over the last twenty years. As our understanding of the role of the gut microbiota advances and our knowledge of the antioxidant and anti-inflammatory functions of polyphenols accumulates, there emerges a need to examine the prebiotic role of dietary polyphenols. This review firstly overviews the importance of the GM in health and disease and then reviews the role of dietary polyphenols on the modulation of the gut microbiota, their metabolites and how they impact on host health benefits. Inter-dependence between the gut microbiota and polyphenol metabolites and the vital balance between the two in maintaining the host gut homeostasis are also discussed.

## 1. Introduction

The human body is a very complex system that is composed of trillions of cells interacting with each other, forming tissues and organs. It includes an even greater number of bacteria that inhabit our skin, the epithelium of the mouth, nose, vagina and, above all, the digestive system. The term “superorganism” has been used to describe the entire interactive complex of cells of the human body, its microsystems and microbiota. In 2001, Joshua Lederberg defined the concept of the microbiome as the ecological community of commensal, symbiotic and pathogenic microorganisms that literally share a body space [1]. It has been found that bacteria constitute 1–2% of the human body weight, and it is estimated that the human body (70 kg) may be colonized by as many as 3.8 × 10^13^ microbial cells. The number of bacteria in the body is actually of the same order as the number of human cells [2].

Metagenome analysis shows that the amount of unique genes of bacteria inhabiting the human body is about 3.3 million, so about 150 times the number of our own genes. Such a large number of genes translates into a huge potential, e.g., metabolic. This confirms the need for a holistic view of the human body in which the microbiome can give it features that it would not be able to develop by itself [3,4].

The growing scientific interest in the composition of the microbiome and its impact on human health has led to the initiation of long-term projects in this topic. They include the NIH Human Microbiome Project, ELDERMET, Microbes and MetaHIT projects [5].

There is a diversity of microbes, and the vast majority of microbes inhabit our gastrointestinal tract, with the greatest number residing in the distal gut [6]. Diet is considered to be one of the main factors influencing the development of the intestinal microflora throughout life [7]. Other factors include the environment, lifestyle and genetic predisposition [6,8]. Gut bacteria play a crucial role in the maintenance of immune and metabolic homeostasis and protection against pathogens. Indeed, certain considerable changes in the composition of the gastrointestinal microflora (dysbiosis) may be associated with the pathogenesis of various diseases [7]. There are opportunities to improve human health through monitoring or manipulation of the human gut microbiome [1].

This review aims to update the knowledge and highlight the impact of polyphenols in modulating the gut microbiota and its impact on the prevention and course of metabolic syndrome (MetS).

## 2. Metabolic Syndrome

Metabolic syndrome (MetS) has been described as the coexistence of multiplex and interrelated risk factors for atherosclerotic cardiovascular disease (ASCVD) and type 2 diabetes [9]. MetS also includes co-occurrence of insulin resistance, hyperinsulinism, impaired glucose tolerance, obesity, hypertension, pro-thrombotic state and atherogenic dyslipidemia [10].

According to contemporary research, MetS has been associated with many other clinical conditions such as oxidative stress, pro-inflammatory state, hepatosteatosis and non-alcoholic fatty liver disease, hyperandrogenism, polycystic ovary syndrome, obstructive sleep apnea, vascular dementia and Alzheimer’s disease [11,12]. Studies have demonstrated a link between the number of metabolic syndrome components and increased risk of (also sudden) cardiac death [13]. MetS or its components have been linked with cancer development (i.e., colon-rectal and pancreatic) and mortality [14].

MetS was first defined in 1998, by the World Health Organization [15]. However, various organizations have proposed different diagnostic criteria over the past decade. In an attempt to harmonize the criteria, a set of risk factors was established, and three risk factors out of the five listed would qualify a person for metabolic syndrome. These include:

- Elevated waist circumference;
- Elevated triglycerides (≥150 mg/dL; also during pharmacotherapy);
- Reduced high-density lipoprotein (HDL) cholesterol (<40 mg/dL; drug treatment for reduced HDL cholesterol is an alternate indicator);
- Elevated blood pressure (systolic ≥ 130 and/or diastolic ≥ 85 mm Hg; also during antihypertensive pharmacotherapy);
- Elevated fasting glucose (≥100 mg/dL; pharmacotherapy of incorrect level of glucose is an alternate indicator) [16,17].

All the factors that make up metabolic syndrome influence one another. Simply put, dependents such as obesity and a lack of exercise lead to the development of insulin resistance. Insulin resistance increases the concentration of low-density lipoprotein (LDL) cholesterol and triglycerides and reduces the concentration of HDL cholesterol in the blood serum. This can lead to fatty plaque deposits in the arteries, which, over time, can cause cardiovascular disease, blood clots and strokes. Insulin resistance also increases the concentration of insulin and glucose in the blood serum. Chronic elevated glucose levels, in turn, damage blood vessels and kidneys, which can lead to type 2 diabetes. Moreover, an abnormal insulin level mediated by sodium concentration could lead to high blood pressure. It should be noted that the mutual relations between the individual components of the syndrome are not of the same intensity, but the body mass index correlates with all other components [12,18].

Patients suffering from metabolic syndrome require greater utilization of medical care compared to people without metabolic syndrome. The increase in health care expenditure is approximately 20% for each additional risk factor [11]. MetS is common, ranging in prevalence from 10% to 40% of the world’s population [10].

In accordance with Cameron et al. and Saklayen [19,20], there are currently insufficient global data strictly on metabolic syndrome. This may result, inter alia, from the use of different classification criteria; hence, it is important to establish standard methods and criteria for the diagnosis of the syndrome in order to improve the accuracy of future standardized comparative tests. According to the APTIII criterion (National Cholesterol Education Program Adult Treatment Panel III), the prevalence rate for individual countries is, respectively: United Arab Emirates—36.9%, North Jordan—36.6%, Urban Indians—35.2%, United States—34%, Iran—33.7%, Brazil—29.8%, Italy—27.2%, Turkey—26.9%, Greece—24.5%, Portugal—24%, The Netherlands—23%, Slovakia—20.1%, Thailand—20.0%, Hungary—8.3%, Spain—5% [21].

The results of statistical studies suggest that even within the same ethnic group there may be significant differences in the occurrence of both the individual factors constituting metabolic syndrome and metabolic syndrome itself [19]. However, it was found that lower educational levels were significantly associated with metabolic syndrome. Generally, the prevalence of metabolic syndrome increases with age, and hence the results obtained for individual countries may be related to the age structure of the population. It was verified that the prevalence of metabolic syndrome is often greater in the urban population of some developing countries than in their Western counterparts. This state of affairs is caused by an increase in the consumption of high-calorie and low-fiber fast food, as well as a decrease in physical activity as a result of sedentary work and leisure activities, and the use of mechanized transportation [20].

All these aspects make MetS one of the significant current health and clinical challenges worldwide [11,22].

## 3. Healthy Microbiota Composition and Functions

Microorganisms colonize all surfaces of the human body, but the gut is the site of a particularly rich microbiome. The gut is characterized by an ecological diversity of microorganisms—with more than 100 trillion microbial cells living symbiotically within it [23]. The set of gut microbiota (GM)—bacteria, archaea, viruses, eukaryotes and other microbial species—has evolved jointly with the host over millions of years, forming a complex and symbiotic connection [24]. Microbial colonization in the gastrointestinal tract starts immediately after birth.

The intestinal microflora content changes along the gastrointestinal tract (GI). In the initial proximal part of the small intestine, it is very similar to the composition of the gastric microbiota [24]. The differentiation and accumulation of bacteria increase in the distal part, from the duodenum to the ileum, with increasing pH. This part is dominated by *Lactobacillus*, *Clostridium* and *Escherichia coli* species, as well as the Bacteroidetes phylum and Gram-negative anaerobes [25]. Conditions in the large intestine are favorable to bacterial growth and allow for a more diverse and complex bacterial collective, consisting mainly of obligate anaerobes. In the colon, the main bacteria are *Ruminococcus*, *Lactobacillus* and *Clostridium* species (Firmicutes phylum), as well as *Bacteroides* and *Prevotella* (Bacteroidetes phylum). It is worth emphasizing that Bacteroidetes and Firmicutes phyla account for over 90% of the complete bacterial community in the gut [26]. The diversity of the GM grows with human development. A complex and stable microbiome is formed at about 3 years of age.

It has been reported that the composition of the human gut microbiota changes with age. The diet of elderly individuals changes for many reasons (e.g., loss of dentition, impaired salivary function, digestion and transit time in the GI tract, as well as certain diseases). However, the composition of the intestinal microbiota may also vary in elderly subjects independently from diet because of immunological and physiological agents. The GM associated with elderly individuals is characterized by a reduced abundance of *Ruminococcus* and *Blautia* spp. and a diminished abundance of several butyrate producers, while the number of *Escherichia* is increased compared with young controls. The ratio of *Firmicutes* over *Bacteroidetes* changes throughout the lifespan: it is lower in the first year of life (0.4), increases in adulthood (10.9) and decreases in old age (0.6) [5].

Odamaki et al. [27] investigated the sequential changes in gut microbiota composition in newborn to centenarian Japanese subjects. The results indicated some patterns and transition points in gut microbiota composition with age. The transition from infant to centenarian was accompanied by distinctive changes in the abundance of *Bacteroides* (elderly-associated), *Enterobacteriaceae* (infant- and elderly-associated), *Bifidobacterium* (infant/child-associated) and *Lachnospiraceae* (adult-associated). *Megamonas* and *Peptoniphilus* were relatively enriched in the elderly. *Dorea* abundance appeared unrelated to aging. Given the age-related reduction in the abundance of the genus *Bifidobacterium*, which down-regulates pro-inflammatory responses in the gut epithelium, the results suggested that the aging-related dysbiosis in elderly subjects may be a contributing factor to inflammatory responses that occur with advancing age.

A lot of results suggested that nutrients in the gut might play an important role in changing the GM composition with age. It was found that dietary supplementation with a branched-chain amino acid (BCAA) exerts a variety of beneficial effects in mice and humans. In mice, BCAA supplementation can promote longevity, but its influence on the gut ecosystem and the underlying mechanism remain unclear. The results showed that the structure of the gut microbiota changed, and BCAA supplementation in mice slowed the change speed of the gut microbiota which is due to age. In addition, the abundance of *Akkermansia* and *Bifidobacterium* increased in BCAA-supplemented mice, while the ratio of *Enterobacteriaceae* decreased in BCAA-supplemented mice. Moreover, many metabolites, representing sugar and lipid metabolism, were altered between the supplemented and control groups. Thus, BCAAs influence the gut microbiota and gut metabolism. In addition, the BCAA-supplemented group presented lower serum concentrations of lipopolysaccharide-binding protein. The changes are indicative of lower antigen loads in the host gut. These results suggest that dietary supplementation with BCAAs may be considered for improving health and promoting healthy aging [28].

Further analyses investigating lifestyle traits or prospective cohorts focused on subjects who appear to have a gut microbiome typical of an age group older than their matched age would be valuable for revealing the relationships between the gut microbiota and host health, including the aging process.

The intestinal microbiome plays a key role in supporting the immune and metabolic functions of organs and tissues [29]. Bacteria participate in various metabolic functions, such as enzymatic digestion, fermentation and absorption of complex carbohydrates and dietary proteins [30]. The GM produces several bioactive compounds, some of which are beneficial to health, e.g., vitamins and some short-chain fatty acids (SCFAs), while others are deleterious, such as some metabolites of degradation of amino acids. In addition, the intestinal bacteria have the ability to synthesize essential and nonessential amino acids [20]. The GM makes key contributions to the metabolism of xenobiotics, transforming dietary components and pharmaceuticals into metabolites with altered activities, toxicities and lifetimes within the body [31]. Microbial metabolism of xenobiotics must be understood in the context of the concurrent and often competing metabolic processes occurring in the human host. Orally ingested compounds pass through the upper gastrointestinal tract to the small intestine where they can be modified by digestive enzymes and absorbed by host tissues. Readily absorbed xenobiotics pass between or through intestinal epithelial cells, where they may be processed by host enzymes before transport to the liver via the portal vein. Following exposure to the liver’s rich collection of metabolic enzymes, xenobiotics and their metabolites enter the systemic circulation, distributing into tissues and potentially affecting distal organs. However, intravenously administered compounds circumvent this “first-pass” metabolism and are immediately introduced into the systemic circulation. In contrast to compounds that are absorbed in the small intestine, poorly absorbed xenobiotics continue through the small intestine into the large intestine and may be transformed by gut microbes. The products of gut microbial metabolism can be absorbed by the host and circulated systemically or interact locally with the epithelial cells lining the gastrointestinal tract. Ultimately, these microbial metabolites are excreted in feces or filtered by the kidneys and eliminated in the urine. In conclusion, these processes generate a complex intertwined metabolic network that affects both the host and the members of the microbiota [2,32].

Bacteria of the gastrointestinal tract also constitute a physical barrier that protects the human organism against pathogenic bacteria [20]. Moreover, the microbiome exerts comprehensive defense functions against pathobionts, supporting the integrity and regulating the permeability of the intestinal barrier. Thus, it contributes to the maintenance of homeostasis in the host entity [33].

The colon microbiome also converts primary bile acids that are not reabsorbed into secondary bile acids (deoxycholic and itocholic acids, and others). It should be noted that the composition of the colon microbiome has an effect upon the amount of secondary bile acids produced [34]. Bile acids play an important role in many metabolic diseases. The absorption of lipids and vitamins is facilitated by bile acids. Furthermore, they support intestinal motility and regeneration of the liver. In addition, they reduce inflammatory processes. These compounds exert their effects through the farnesoid X receptor (FXR) and the TGR5 receptor, which are present in many cells and tissues. Beyond the aforementioned, bile acid-induced stimulation of TGR5 reduces cytokine production. In contrast, the binding of these substances to FXR improves insulin sensitivity, inhibits hepatic gluconeogenesis and lowers lipid levels [23].

Moreover, the gut microbiota can metabolize the complex oligosaccharides that are not digested. By means of these processes, it becomes possible to recover absorbable substances; hence, the microbiota have the ability to increase the obtaining of energy and nutrients from food [33]. In addition to the above, the microbiota play an important role in maintaining the host’s immune system. Numerous studies emphasize the immense role of the microbiome in the functioning of lymphoid tissues and lymphocytes associated with the intestine [35]. In a healthy host, the GM generates favorable responses to pathogens while maintaining tolerance to safe antigens. Compositional and functional changes of the gut microbiota have been reported in various autoimmune diseases (AIDs), and increasing evidence suggests that a disturbed gut microbiota contributes to their immunopathogenesis. Mechanisms include abnormal microbial translocation, molecular mimicry and dysregulation of both local and systemic immunity [36]. In recent years, the gut microbiota of patients with AIDs, including rheumatoid arthritis, systemic lupus erythematosus [37], spondyloarthritis primary Sjögren’s syndrome and Behcet’s disease [38], was found to be significantly different from that of healthy people. Species that colonize the human gut, such as *Prevotella copri*, *Ruminococcus gnavus* and *Lactobacillus salivarius*, are implicated in the pathogenesis of AIDs. Although different animal models indicate the immunomodulating activity of certain bacterial strains and their metabolites [39], understanding the clinical implications of these findings remains challenging.

The intestinal microbiome has an influence upon distant organs, for example, by the production of short-chain fatty acids, lipopolysaccharides, bile acids and peptidoglycans, as well as trimethylamine (TMA) and trimethylamine N-oxide (TMAO) [40]. TMA is made by the GM after meals containing choline or phosphatidylcholine or carnitine. These compounds are contained in foods high in fat. Humans lack TMA lyases, so all TMA is produced by the gut microflora. The absorbed TMA is transported to the liver where flavin monooxygenase-3 oxidizes the TMA to TMAO [34]. Elevated levels of trimethylamine N-oxide in the blood are seen as one of the causes of atherosclerosis [41]. Numerous studies have demonstrated that SCFAs play an important role in combating metabolic diseases. They exert a significant influence on the formation of atherosclerotic plaques by improving the function of the intestinal barrier. Moreover, short-chain fatty acids can modulate the immune and inflammatory response by activating free fatty acid receptors (FFARs) and inhibiting histone deacetylases [42]. The binding of SCFAs to receptors can also induce stimulation and suppression of inflammatory cytokine production, as well as influencing the migration and recruitment of immune cells into atherosclerotic plaques.

Numerous pieces of evidence suggest that the GM plays an important role in the gut–brain axis (GBA) structure. The GBA is a bidirectional link between the central nervous system and the enteric nervous system. It involves direct and indirect pathways between cognitive and emotional centers in the brain with peripheral intestinal functions. The GBA involves complex crosstalk between the endocrine (hypothalamic–pituitary–adrenal axis), immune (cytokine and chemokines) and autonomic nervous systems [43].

Many studies have shown that the gut microbiota produces neurotransmitters. *Lactobacillus* and *Bifidobacterium* can produce gamma-aminobutyric acid, which is the main inhibitory neurotransmitter in the brain. In addition, *Candida*, *Escherichia* and *Enterococcus* produce serotonin. The GM also plays an important role in tryptophan metabolism which is the precursor to the production of serotonin. Moreover, bacterial products are known to stimulate enteroendocrine cells to produce neuropeptides, which enter the bloodstream or directly influence the enteric nervous system [43]. Many researchers try to explain how the GM can influence neuropsychiatric disorders. Asano et al. [44] showed that *Clostridium* produced biologically active dopamine in the gut lumen of mice. Given that dopamine is the key neurotransmitter associated with schizophrenia, it is possible that these bacterial metabolites can interact and stimulate the central and peripheral nervous systems. The study by Desbonnet et al. [45] showed that rats that had undergone maternal separation (model of induced depression-like behavior) could be rescued by treatment with the probiotic *Bifidobacterium infantis* in conjunction with citalopram but not when they were administered separately. Meanwhile, clinical studies on subjects with schizophrenia showed the presence of increased levels of lactic acid bacteria in the gut lumen, including *Lactobacillus casei*, and *Lactobacillus lactis* as well as *Streptococci species* such as *Streptococcus mutans* and *Streptococcus thermophilius* [46]. The increased presence of these bacteria species is associated with alterations in adaptive Th2 immune responses which are known to be present in schizophrenia. Administration of probiotics to these individuals altered the microbiome and appeared to normalize some behavioral symptoms.

## 4. Dysbiosis and Metabolic Syndrome

The term “dysbiosis” was first used in the mid-20th century to depict the changes in intestinal bacteria, suggesting a link with immune homeostasis impairment and development of intestinal disorders. In general, dysbiosis is characterized by a decrease in the number of symbionts, an unwarranted growth of pathobionts and/or a loss of their diversity. These different factors can coexist, which is most often the case. Several factors are responsible for dysbiosis, including age, diet and lack of exercise, stress, drugs and xenobiotics [47]. Moreover, demographic dietary factors result in differential variation in the GM. Rural African children, usually consuming plant polysaccharides, had lower fecal levels of *Firmicutes* and higher fecal levels of *Bacteroidetes* when compared with Italian kids that present high amounts of *Enterobacteriaceae* [48]. Several pieces of evidence from preclinical models and from human studies consistently show that high-fat and high-sugar diets modify the GM profile towards dysbiosis, while a vegan diet, prebiotics and probiotics cause beneficial effects on GM composition and function, accompanied by reduced adiposity and inflammatory molecules, including lipopolysaccharides, and other positive effects on metabolic hemostasis [47]. Other research indicates the involvement of GM dysbiosis in gastrointestinal and extraintestinal metabolic diseases, such as obesity, type 2 diabetes and cardiovascular diseases [6].

Dysbiosis triggers the breakdown of the intestinal barrier integrity and modifies the expression of tight proteins. As a result, intestinal permeability increases, and, as a consequence, bacterial fragments and uremic toxins move into the bloodstream. This process causes endotoxemia, i.e., low-grade inflammation. Pro-inflammatory molecules are released, which adversely affects glucose and insulin metabolism [24,49]. This is further driven by glycation products and other oxidative pathways that are involved in metabolic deficiencies in the development of obesity and diabetes.

At the same time, signals from the dysbiotic microbiome modulate the immunometabolic effect on epithelial cells and the immune system. The resulting immune-inflammatory environment favors the progression of diabetes and its complications [50]. For this reason, dysbiosis also contributes to the progression of some of the major microvascular complications of diabetes. It has been suggested that an increase in circulating bacterial endotoxins, especially lipopolysaccharides, may play a significant role in the low-grade inflammation associated with obesity, diabetes and microvascular complications. The movement of bacterial components across an impaired intestinal barrier into the systemic circulation can also produce the inflammation characteristic of diabetic retinopathy and nephropathy [50]. Overall, these mechanisms may play a decisive role in the development of the metabolic and vascular complications of diabetes.

## 5. Classes of Phenolic Compounds and Their Biological Role for Human Health

### 5.1. Structure and Classification of Phenolic Compounds in Plants

Polyphenols, classified as secondary plant metabolites, constitute a diverse group of compounds, the common feature of which is the presence of at least two hydroxyl groups attached to one or more aromatic rings. They are a class of non-essential phytonutrients and are found in various plant parts, such as stems, leaves, flowers, roots and pulp [51]. It has been documented that phenolic compounds may be conjugated with sugars or organic acids or as polymers and interact with proteins. The elementary structure of polyphenols is an aglycone, but they are predominantly distributed as glycosides in plants [52]. More than 8000 natural polyphenol structures have been identified [53].

**Figure 1:**
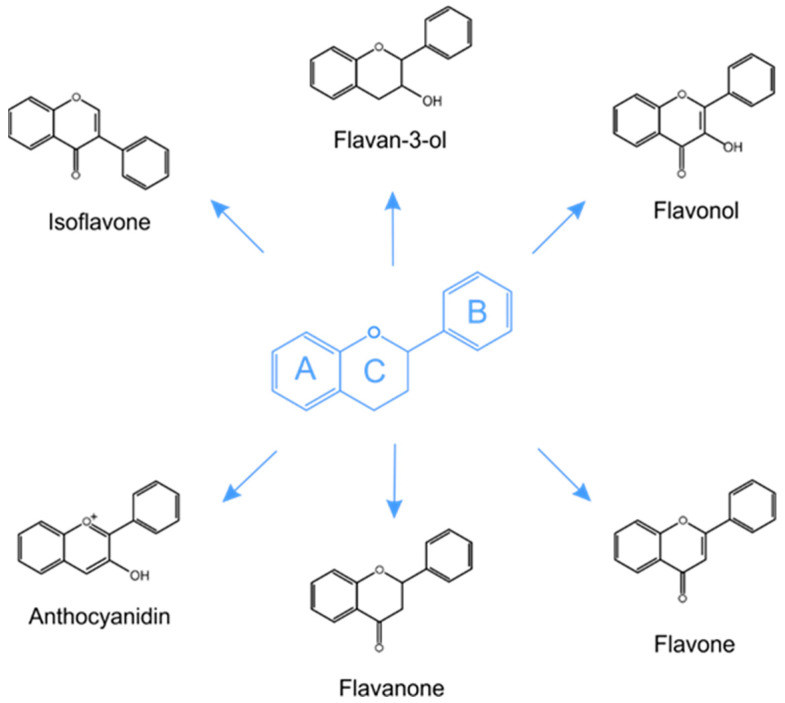
Generic structure of major flavonoids.

The most common classification embraces the subdivision of phenolic compounds into two main groups: flavonoid (e.g., anthocyanins, flavanols, flavanones, flavonols, flavonones and isoflavones) and non-flavonoid (e.g., phenolic acids, xanthones, stilbens, lignans and tannins) polyphenols [54,55]. The basic flavonoid molecule is made up of 15 carbon atoms that form two benzene rings (Figure 1). Between them is a three-carbon structure in the form of a heterocyclic ring of pyran or pyron. Anthocyanins are water-soluble flavonoids responsible for the red-orange to blue-violet color of fruits and flowers. The basic structural unit of anthocyanins is the flavylium ion (2-phenylchromenylium). The obligatory structure of phenolic acids includes a carboxyl group linked to a benzene ring. Derivatives of benzoic (e.g., gallic, vanillic, syringic) and cinnamic (e.g., sinapic, caffeic, *p*-coumaric) acids are possible [54].

Polyphenolic compounds have been subjected to much research. This is because they display a number of properties that are beneficial to health, and offer specific therapeutic potential. In most plant materials, they appear as compounds accompanying other active substances. Hence, the possible synergism of their action must be taken into account. Due to the ability to inactivate or prevent the formation of reactive free radicals, polyphenolic compounds exhibit antioxidant properties. The antioxidant properties of polyphenols are determined by their chemical structure, more specifically by the presence of hydroxyl groups. The strength of this action depends on their number and position in the ring structure, esterification or proximity to other substituents [56,57].

### 5.2. Polyphenolic Compounds as an Important Plant Component of the Diet

Polyphenols are abundant micronutrients in a balanced healthy diet. They are mainly eaten as ingredients in vegetables, fruits and cereals [58]. Anthocyanins are present in very large amounts in some diets. The valuable and richest sources of this class of polyphenols are dark-colored fruits and vegetables (black elderberries, black chokeberries, cherry, black olive, red lettuce, red onions) and specific varieties of colored ones (orange juice, goosegogs). There are especially many of such compounds as malvidin 3-glucoside, cyan 3-glucoside and elargonidin 3-glucoside [59]. The main sources of flavanols are cocoa, dark chocolate, black chokeberries, blueberries, blackcurrant, strawberries and apples. Hazelnuts and pecan nuts, as well as pistachios and other almonds, are other relevant sources [58]. (+)-Catechin and (−)-epicatechin are commonly found in plant foods [60]. Black tea, green tea and red wine contain high levels of flavanols, particularly catechins (epigallocatechin, 4′-O-methyl-epigallocatechin) and proanthocyanidin dimers [58,61]. Products that are high in flavonols are cranberries, onions, green tea and red wine. Among flavonols, particularly quercetin, myricetin and kaempferol have been extensively studied, mainly because of their wide distribution in dietary plants [61]. Phenolic acids are highly distributed among plants. They are present in the composition of, e.g., anise, cumin, curcumin (used as spices), wild blueberries, coffee, carrots, plums, aubergines, tomatoes and cereals [58]. Hydroxybenzoic acids are typically found in berries at low concentrations. Protocatechuic acid, *p*-hydroxybenzoic acid and gallic acid are present in the composition of blackberries, red raspberries and sweet cherries. Overall, *p*-hydroxybenzoic acid and protocatechuic acid are the dominating hydroxybenzoic acids in ripe fruits. Quinic acid esters are mainly found in blueberries, kiwis and cherries. Uniquely high amounts of chlorogenic acids are found in green coffee [60]. One of the main food sources of ferulic acid is wheat bran [62]. Isoflavones are provided by soybean-derived products. Some of the most important examples of this group of compounds found in soybeans are daidzin and genistin [58].

Dietary Reference Intake (DRI) values exist for vitamins and minerals and provide a guideline on the optimal dose range to avoid deficiency and prevent toxicity [63]. Many epidemiological studies support the action of polyphenols or polyphenol-rich foods on health, but the topic still has a lot to explore. More adequately powered, randomized, placebo-controlled human studies are needed on polyphenols. There is a large number of structurally different polyphenols which are relevant for health, and obtaining enough information to set a DRI for each of these will not be feasible in the foreseeable future. Nowadays, a target intake value of polyphenols as “lifespan essentials” needs to be based on the amount of polyphenols in “5-a-day” (it is estimated at 0.5–1 g). Data on the consumption of flavonoids in the diet in Western countries suggest 50–800 mg, while in Eastern countries, consumption is even 2 g (as a diet rich in fruits and vegetables) [64]. It has been estimated that the average person eats 100 to 1000 mg of polyphenols per day, but an accurate subgroup estimate is not straightforward due to the very different dietary traditions between different nationalities [64]. According to data published in an article in 2021, total polyphenol averaged intake for the general population was estimated to be 0.9 g per day, where the main dietary founts were coffee, tea, wine, fruits and vegetables [61].

Epidemiological studies have suggested associations between the consumption of polyphenol-rich foods or beverages and the prevention of diseases [65]. Deficiency of phenolic compounds in long-term diet can increase the risk of many diseases, such as certain cancers, cardiovascular diseases, type 2 diabetes, osteoporosis, pancreatitis, gastrointestinal problems, lung damage and neurodegenerative diseases [66]. On the other hand, some adverse effects have been reported from polyphenol intake [66]. Polyphenols from the daily diet can affect the transport of thiamine and folic acid, as well as changing the activity of drugs through interactions that affect drug transporters or enzymes involved in reactions, resulting in both inhibition and increasing bioavailability depending on the case. This is due to, inter alia, pre-absorptive interactions during digestion [67]. Polyphenols chelate iron, which can result in iron absorption inhibition and iron status deficiency [68]. Proanthocyanidins and ellagitannins have been considered antinutritional compounds because of their ability to interact with proteins and inhibit several enzymes [69]. Isoflavones may impact the long-term growth and pubertal development of children fed soy-based formulas in infancy [66,70]. Earlier studies have suggested that isoflavones may adversely affect the development of estrogen-sensitive breast cancer and endometrial cancer. This was explained by the fact that these compounds are characterized by endocrine-disrupting properties. Significantly, recent epidemiological reviews suggest either a null or protective effect of isoflavones on these cancer types [66]. There are suggestions that some polyphenols may have carcinogenic or genotoxic effects at high doses or concentrations. As an example of such information, caffeic acid (dietary content was 2%) induced forestomach and kidney tumors in rats and mice [71]. Notwithstanding, most of these effects have been shown in in vitro or animal studies, and it has not been proved that these effects also occur among humans. It is also important that the food matrix may also influence the effects of polyphenols and the doses applied in experiments have been large [72].

Polyphenolic compounds interact with food macronutrients. Interactions of polyphenols with plasma proteins (albumin is the primary protein that interacts with polyphenols in the blood, especially with quercetin) and food proteins may occur [73]. For example, milk caseins were identified as interfering agents via interaction with tea catechins and the subsequent formation of complexes [74]. Moreover, the binding of proteins with polyphenols reduces the antioxidant capacity. Additionally, the carboxyl and hydroxyl groups of phenolic acids are capable of binding to starch and other polysaccharides. Oligomeric and polymeric procyanidins also have the ability to bind to a variety of polysaccharides [73]. Nearly 35% of the polyphenols in red wine are bound to soluble dietary fiber [75]. However, there are also reports suggesting that the increased absorption of polyphenolic extracts is favored by a meal rich in carbohydrates. Fatty acids may affect the bioavailability, absorption kinetics (also by reabsorption events via the enterohepatic circulation) and antioxidant activity (especially in combination with milk proteins) of polyphenols. The aggregation of the fat globules affects the way that gastrointestinal enzymes access substrates, and also lipase activity is inhibited by phenolic compounds, as well as long-chain fatty acids generated during digestion [73].

Polyphenols may interact with certain pharmaceutical agents and enhance their biologic effects by affecting drug bioavailability and pharmacokinetics. Consumption of grape juice (rich in naringenin) may cause 3-fold increases in the bioavailability of certain medications, such as benzodiazepines and terfenadine, because of inhibition of CYP3A4. Described actions are clinically significant in the case of cyclosporine because of a narrow therapeutic range (e.g., apply during treatment after organ transplants) [72].

### 5.3. Health Benefit Properties of Plant Polyphenolic Compounds

Plant polyphenols have noted anti-inflammatory and analgesic activity. These compounds have the ability to interfere with cellular signaling pathways, can express numerous genes and can modulate the activity of pro-inflammatory enzymes and cytokines. Some flavonoids and phenolic acids can affect the activity of enzymes related to the synthesis of inflammatory mediators (prostanoids, leukotrienes or NO), including COX-2 cyclooxygenase and induced NO synthase. This anti-inflammatory activity is related to an effect on cells that belong to the body’s immune system. It has been reported that polyphenolic compounds (e.g., quercetin or naringenin) can influence the expression of cell surface receptors, as well as the activity of cells involved in the response to allergens. Moreover, polyphenols may indirectly influence the function of the immune system by modulating the composition and activity of the microbiome inhabiting the gastrointestinal tract [76,77,78].

An example of the practical application of the immunomodulatory properties of polyphenols is wound healing therapy. An excessive reactive oxygen species (ROS) amount is one of the leading causes of impaired chronic wound healing. The beneficial effects of these compounds are based on their antioxidant, anti-inflammatory, profibrogenic and proangiogenic properties [79,80].

Antibodies produced by cells of the immune system protect the body against various infections and diseases, but in pathological conditions, they can cause complex autoimmune disorders. Due to the troublesome side effects and often unsatisfactory therapeutic effect of conventional pharmacotherapy, research is being conducted on the possibility of including polyphenolic compounds in the therapy of autoimmune diseases. In this case, their antioxidant and anti-inflammatory properties are emphasized. In the light of research results, they seem to be a promising alternative therapy for diseases such as ulcerative colitis, leucoderma, rheumatoid arthritis, sarcoidosis and multiple sclerosis [81,82].

Furthermore, polyphenolic compounds can inhibit neurodegenerative processes. Such action is related to, e.g., the ability to modulate mitochondrial dysfunction, to regulate antioxidant and anti-inflammatory agents (glutathione, cytokines, superoxide dismutase) and to reduce oxidative stress. Polyphenols also demonstrate the ability to inhibit the activity of cholinesterase, which is one of the most important therapeutic strategies for Alzheimer’s disease [83,84,85].

New possibilities of using flavonoids and phenolic acids in the treatment of epilepsy are being sought because they have many significant biological and medicinal properties, including antioxidant, anti-inflammatory and neuroprotective activity. These effects seemed to be mediated by two primary mechanisms: by the alleviation of hippocampal structural changes, including through granule cell dispersion in the dentate gyrus, and by the reduction in inflammatory responses, which is well represented in temporal lobe epilepsy, by inhibiting the expression of pro-inflammatory cytokines in kainic acid-induced seizure model mice [86].

Furthermore, the use of polyphenols in the treatment of Parkinson’s disease without the common side effects of traditional drugs has been researched [87]. In this context, antioxidant and anti-inflammatory properties, but also the ability to modulate protein misfolding of polyphenols, are crucial. Notably, polyphenols protect neurons from reactive oxygen species by increasing the activity of NAD-dependent deacetylase sirtuin-1 (SIRT1) and dampen the neuroinflammatory process by modulating the signaling of nuclear factor kappa-light-chain-enhancer of activated B cells. In addition, PPH can induce neurite outgrowth, increase cellular antioxidant defense and regulate pro-survival transcription factors and gene expression. It is worth mentioning that polyphenolic compounds reduce toxicity induced by αSyn misfolded aggregates and amyloid β protein oligomers [88].

Most plants with documented anticancer activity contain polyphenols. The chemoprevention of polyphenolic compounds is based on many complex mechanisms. One of them is their antioxidant activity, which prevents the excessive formation of ROS, which is a procarcinogenic factor. Polyphenols also induce immunomodulation, inhibit angiogenesis and have an influence upon specific actions at the stage of initiation, promotion and progression of carcinogenesis. Moreover, they have an influence upon changes in redox potential in neoplastic cells [54,77,89].

Literature data indicate that plant polyphenols are effective in preventing and inhibiting the growth of gastrointestinal neoplasms, where they are found in the highest amounts after consumption. They are also effective in the case of colorectal neoplasms [51] and contribute to the inhibition of the development of many other neoplasms, e.g., melanoma, prostate and hormone-dependent tumors [54,90]. Furthermore, research indicates that polyphenols, such as apigenin, quercetin, resveratrol or gallic acid, have anti-angiogenetic properties [91].

In the context of cardiovascular diseases, the antioxidant activity of polyphenols has some importance. Diets rich in polyphenols have been found to correlate with reduced morbidity and a milder course of cardiovascular disease. Oxidative stress, inflammation within the blood vessels and endothelial dysfunction have an influence upon the severity of cardiovascular disease, and antioxidants counteract these risk factors. Here, polyphenols inhibit the oxidation of LDL cholesterol-related lipoproteins and platelet aggregation. Beyond the aforementioned, polyphenolic compounds show antiatherosclerotic, antiarrhythmic and vasodilating effects. Moreover, research has demonstrated that a diet rich in polyphenols reduces arterial hypertension [54,76,92]. Finally, polyphenols contribute to the prevention of thrombotic episodes [78].

Polyphenols can also have a positive effect on the prevention and alleviation of the course of diabetes. This is because polyphenols can modulate carbohydrate and lipid metabolism, lessen hyperglycemia, dyslipidemia and insulin resistance, improve fat metabolism and alleviate oxidative stress. In addition, polyphenolic compounds can prevent the development of long-term diabetes complications, including cardiovascular disease, neuropathy, nephropathy and retinopathy [93]. Moreover, this group of secondary plant metabolites reduces the risk of obesity [94]. Furthermore, polyphenols exhibit significant activity against adverse microorganisms and viruses [95].

## 6. Metabolism of Polyphenols by Gut Microbiota

The structural complexity and polymerization of polyphenols affect their low absorption in the small intestine (less than 10%) [96]. Their poor bioavailability is, therefore, a major concern for their application as therapeutic agents [23]. The unabsorbed polyphenols accumulate in the large intestine, along with the bile conjugates, and there, they are treated with intestinal microbiome enzymes. Therefore, the GM is extremely important in turning these polyphenols into bioavailable products. The intestinal microbiome metabolizes glycosylated polyphenols into lower-molecular weight phenolic compounds, for example, phenolic acids [97,98].

Biotransformation of polyphenols can occur through various reactions, including hydroxylation, oxidation, decarboxylation, methylation, isomerization, hydration, dehydrogenation and glycosylation [99,100]. Studies conducted by Tao et al. [101] confirmed that the microbiome converts buddleoside flavonoids into their aglycone acacetin forms and then converts aglycone metabolites into hydroxylated, methylated, acetylated and hydrogenated by-products [101]. Methylation of flavonoids might result not only in a dramatic increase in their hepatic metabolic stability but also in great improvement of their intestinal absorption, both of which could greatly increase their oral bioavailability [102]. This indicates that methylated, hydroxylated, deoxygenated and acetylated metabolites of buddleoside might lead to a higher oral absorption which contributes to improving the bioactivity of buddleoside in vivo. Meanwhile, four strains including *Escherichia* sp. 4, *Escherichia* sp. 34, *Enterococcus* sp. 45 and *Bacillus* sp. 46 showed powerful conversion capability of buddleoside [101].

Studies of the metabolism and intestinal fermentation of mulberry anthocyanins in rats have indicated that the gut microbiome induces the process of decomposition of cyanidin-3-glucoside and cyanidin-3-rutinoside into vanillic, *p*-coumaric, protocatechuic acids and 2,4,6-trihydroxybenzaldehyde, while delphinidin-3-rutinoside is converted to gallic acid, syringic acid and 2,4,6-trihydroxybenzaldehyde [103]. Other results indicate that fermentation of sour cherry polyphenols (chlorogenic and neochlorogenic acids, quercetin-rutinoside and cyanidin-glycosyl-rutinoside) by the gut microbiome produces 4-hydroxyphenylpropionic acids as major metabolites, while 4-hydroxybenzoic acids and epicatechin are also released [104].

Research has revealed that the microbial transformation of ellagitanins releases ellagic acid in the intestinal lumen through hydrolysis after consumption of ellagitanin-rich fruits (pomegranates, strawberries, raspberries). Ellagic acid is then metabolized in the large intestine by the GM (*Gordonibacter urolithinfaciens*, *Gordonibacter pamelaeae* and *Ellagibacter isourolithinifaciens*) to urolithins derivatives. These compounds can either be absorbed during first-pass Phase II metabolism or excreted in the feces. Urolithins have biological activity and may exert beneficial effects on human health [105]. The conversion of ellagitannin and ellagic acid into urolithin is characterized by high interindividual variability in humans, which is related to differences in the intestinal microflora.

Research into *Lactobacillus acidophilus* has indicated that it converts dietary glycosides into aglycones that can be modified by other bacterial species or directly consumed by the host organism [106]. Based on a novel experiment, consumption of red wine polyphenols in combination with the *Lactobacillus plantarum* strain maintained a normal GM content. This in vitro study shows that the initial addition of red wine polyphenols exerted an overall antimicrobial effect on the GM, but the effect disappeared during continuous feeding with polyphenols, probably due to the onset of microbial metabolism of polyphenols. These results indicate a more pronounced effect of the polyphenol compounds on the microbial functionality than on viability [107]. It has also been shown that daidzein, the main soy isoflavone, is metabolized by the gut microbiota to equol, which shows higher anti-estrogenic activity, antioxidant capacity and anticancer effects than daidzein [108]. Three strains of bacteria, the Gram-negative *Bacteroides ovatus* spp. and the Gram-positive *Strepotococcus intermedius* spp. and *Ruminococcus productus* spp., have been identified by culturing the fecal flora from healthy Japanese adults after consumption of tofu and reported to be able to convert pure daidzein to equol in vitro [109]. Moreover, daidzein can be converted by the GM to O-desmethylangolensin (O-DMA). Approximately 80–95% and 25–60% of individuals harbor gut microbial communities capable of producing O-DMA or equol, respectively. Bacteria confirmed to metabolize daidzein to O-DMA in vitro include *Eubacterium ramulus* [110] and *Clostridrium* sp. HGH 136 [111]. In addition, a genus from the *Clostridium cluster* XIVa, named strain SY8519, was isolated from a human fecal sample and identified to also produce O-DMA [112].

The studies described above suggest that the gut microbiota is particularly important in the metabolism of polyphenols into essential bioactive compounds. Based on this research, it can be concluded that the metabolites formed from the degradation of plant polyphenols are mainly responsible for the health-beneficial effects on the host [29].

## 7. Effect of Polyphenols on the Composition of Gut Microbiota

**Figure 2:**
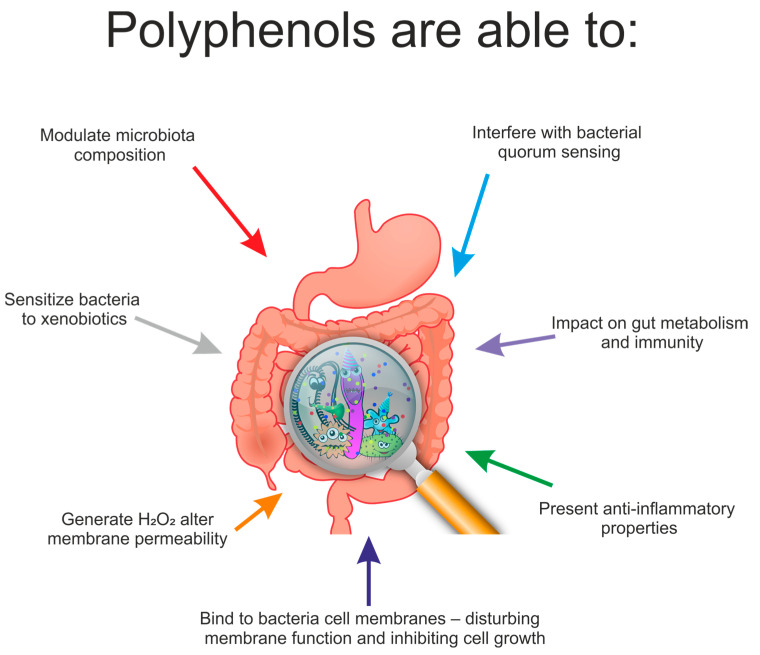
Effect of polyphenols on gut microbiota.

Unabsorbed polyphenolic compounds and produced metabolites that reach the colon may interact with the gut microflora and may modulate the microbial composition through various mechanisms of action (Figure 2).

Research has indicated that polyphenols can modify the ecology of the GM by employing antimicrobial activity or prebiotic-like action against harmful gut bacteria residing in the gut [99,100,113,114]. Research has indicated that lowering the *Firmicutes/Bacteroidetes* ratio and inducing the colonization of certain beneficial bacterial species can provide protection against some health pathologies [29,115]. Gram-positive bacteria are more prompted to the action of polyphenols than Gram-negative bacteria, conceivably due to the differences in the composition of their cell walls [100]. Polyphenols expand the population of profitable species such as *Bifidobacterium* and *Lactobacillus*, which contribute to the protection of the intestinal barrier. Additionally, they can increase the content of *Faecalibacterium prausnitzii*, characterized by an anti-inflammatory effect, and *Roseburia* sp., which produces butyrate [29]. Research confirms the influence of polyphenols on the content of *Akkermansia muciniphila* in the GM. They exhibit a beneficial anti-inflammatory effect and a positive effect in preventing obesity. The population of *Akkermansia muciniphila* in healthy people is at a normal level, but it decreases in patients with active inflammation, or who suffer from diabetes, gastrointestinal diseases and obesity [116].

Metabolites generated during interactions between polyphenols and the GM may be a factor conditioning bacteria multiplication. It must be stressed that this effect can be bidirectional, i.e., they can be either activators or inhibitors of intestinal microbiota growth [117]. Research conducted by Stead [118] showed that hydroxycinnamic, caffeic, coumaric and ferulic acids inhibit the development of *Lactobacillus brevis* bacteria, at a concentration of 0.5 mg/mL. Experiments conducted by Parkar et al. [119] proved that chlorogenic and caffeic acids, at the concentration of 0.25 mg/mL, inhibit the growth of *Lactobacillus rhamnosus* bacteria. Generally, lactic acid bacteria demonstrate much higher resistance to polyphenols, in comparison to pathogenic microbiota. It has been proven that the decrease in the number of *Lactobacillus plantarum*, *Lactobacillus gasseri* and *Pediococcus pentosaceus*, during a 24-h incubation period, only takes place after a 10–20 mg/mL concentration of polyphenols is administered [119].

The effects of some of the particular polyphenols on GM modulation are shown in Table 1.

Many studies have assessed the effect of polyphenol-rich dietary sources on the gut microbiota composition. For example, the modulation of tea on the gut microbiota has gained much interest in recent years. The most potent inhibitors of microorganism growth are presumably the polyphenolic compounds from green and black tea. It has been shown that the bioactive components of tea (which contain hydrolyzable tannins) can inhibit the growth of many pathogens, including *Helicobacter pylori* [132], *Staphylococcus aureus*, *E. coli* [5], *Salmonella typhimurium* [133], *Listeria monocytogenes*, methicillin-resistant *S. aureus*, *Pseudomonas aeruginosa* and hepatitis C virus [134] (in vitro studies). Green tea polyphenols also exert a stimulating effect on the growth of the population of Firmicutes and Bacteroidetes communities, determined in vitro and in an animal study—bringing about pro-health effects—and can reduce *Firmicutes/Bacteroidetes* and improve *Prevotella/Bacteroides* ratios [135]. In contrast, coffee consumption may increase the metabolic activity of the human intestinal bacterial *Bifidobacterium* spp. [136]. In addition, cocoa consumption caused a significant decrease in the proportion of *Bacteroides*, *Clostridium* and *Staphylococcus* in the feces during an experiment carried out on rats [137]. According to other research, cocoa drinking can lead to increases in human GM *Lactobacillus*, *Bifidobacterium* and *Enterococcus* spp. contents [138].

Human subjects research on pomegranate ellagitannins has demonstrated that these significantly suppress the growth of *Clostridium bacteria* and *S. aurous* [139]. However, another study has found that pomegranate ellagitannin (in addition to punicalagin) may increase the population of *Bifidobacteria* spp. and the *Lactobacillus-Enterococcus* group but has no significant effect on the *rectale-Clostridium coccoides* group and *Clostridium histolyticum* group bacteria [121]. Furthermore, ellagitannins have a stimulating effect on the growth of the population of *Akkermansia muciniphila* [140].

Chacar et al. [141] researched the impact of long-term feeding of rats with phenolic compound-rich grape pomace. After administering the extracts for 14 months, all phenolic compound doses (2.5, 5, 10 and 20 mg/kg for day) eliminated the rise in *Clostridium* as witnessed in the control group, in comparison to younger rats. The study indicated that the gut microbiome in rats is selectively modulated by polyphenols to a healthier phenotype in long-term feeding of rats and might counter the detrimental outcomes of aging on the population of the GM. Furthermore, much research has been published concerning the effects of wine ingestion (containing polyphenols, especially resveratrol) on GM composition. The outcome of this work is that red wine ingestion was found to significantly increase the presence of *Enterococcus, Prevotella*, *Bacteroides*, *Bifidobacterium*, *Bacteroides uniformis*, *Eggerthella lenta* and *Blautia coccoides-E. rectale* groups, while the quantity of *Lactobacillus* spp. was unchanged [142]. In contrast, research has revealed that various kinds of soy products promote the growth of *Lactobacillus*, *Enterococcus* and *Bifidobacterium* spp. [143].

Much research has also been conducted on the effects of fruit polyphenols on the composition of the gut microbiota. For example, it is known that cranberry extract polyphenols increase the abundance of *Akkermansia* species [144], orange juice polyphenols increase the population of *Bifidobacterium* and *Lactobacillus* [145] and blueberry polyphenols alter GM composition, such as the abundance of *Bifidobacterium*, *Proteobacteria*, *Actinobacteria* and *Deferribacteres* [146], while mango pulp polyphenols (in patients with inflammatory bowel disease) alter the beneficial fecal microbial composition by significantly increasing the abundance of *Lactobacillus reuteri*, *Lactobacillus* spp., *Lactobacillus lactis* and *Lactobacillus plantarum*, followed up by an increase in the production of fecal butyric acid [147]. The in vitro anti-Helicobacter pylori activity of citrus polyphenols such as hesperetin, naringenin, poncirin and diosmetin has been demonstrated [148].

To sum up, in recent years, studies have been carried out on the influence of specific and groups of polyphenols, polyphenol-rich plant extracts and various parts of plants on the composition of the gut microbiota. Importantly, changes in the qualitative and quantitative composition of the intestinal microbiota may have a positive impact on potential pro-health gut microbiota-associated benefits and changes in development and disease incidence. Finally, the intestinal microbiota has an impact on the biotransformation of phenolic compounds [29,100,113,114].

## 8. Polyphenols, Gut Microbiota and Metabolic Diseases

Metabolic syndrome results from multiple etiologies such as hyperglycemia, insulin resistance, dyslipidemia and hypertension that increase the risk pathogenesis of obesity, diabetes and cardiovascular events [9,10,11]. Evidence shows that complex interactions between diet and the GM are a valid factor behind the production of chronic low-grade inflammation [99]. This type of inflammation, as well as oxidative stress, is a crucial factor for the underlying MetS associated with chronic disorders.

**Figure 3:**
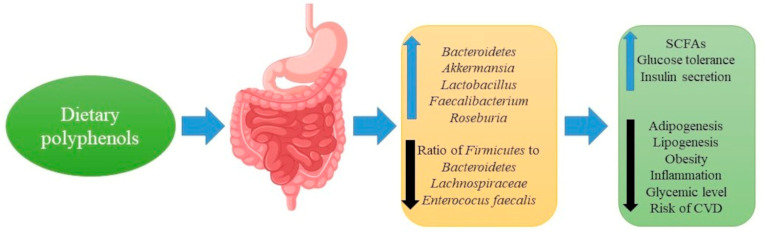
Influence of the dietary polyphenols on the gut microbiota and possible outcome; SCFAs—short-chain fatty acids.

The beneficial effects of polyphenols in the direction of the control of metabolic disorders are a relevant issue that deserves consideration. The mechanism of anti-obesity and antidiabetic effects of polyphenols could be via modulating the microbiota [99] (Figure 3).

Research findings have confirmed that diets rich in polyphenols mediate the increased prevalence of intestinal *Bacteroidetes* species in mice [149], which are associated with the increased capacity of glycan degradation. Moreover, supplementation with polyphenol-rich cranberry extract has been found to prevent high-fructose, high-sucrose diet-instigated weight gain and visceral adiposity formation in mice. Cranberry extract administration has also been found to reduce triglyceride accumulation and to inhibit hepatic inflammation and thereby improve insulin sensitivity. In high-fat diet-fed mice, dietary supplementation of grape polyphenols has been discovered to significantly modulate the gut microbial community structure, such as reducing the *Firmicutes* to *Bacteroidetes* ratio [150].

Moreover, grape polyphenols administration in mice has been noted to promote the growth of *Akkermansia muciniphila*, and these changes have been found to protect against negative consequences associated with high-fat diet [151]. In addition, procyanidin supplementation was reported to show a protective effect against obesity and its associated risk factors. Administration of this compound reduced body weight gain, improved dyslipidemia and increased energy expenditure The mice treated with procyanidin for 12 weeks had reduced body weight by 7% [152]. The beneficial effect of procyanidin against obesity was discerned to be positively connected with the effect of intestinal microbiome modulation (significantly increasing the β-diversity of the intestinal microbiota and *Bacteroidetes* abundance, and reducing the ratio of *Firmicutes* to *Bacteroidetes*) [152]. An elevation in the *Firmicutes/Bacteroidetes* ratio is a characteristic feature of obesity-driven dysbiosis. Another study has also shown that increased *Lachnospiraceae* spp. were linked with the pathogenesis and progression of obesity and type 2 diabetes in mice [153]. Furthermore, procyanidin supplementation also prompted a decrease in the abundance of *Lachnospiraceae*, indicating that the anti-obesity effect of procyanidin was possible via modulating gut microbiota *Lachnospiraceae* [153]. Procyanidins extracted from the litchi pericarp attenuated hyperlipidemia in mice by regulating several key genes involved in hepatic lipid homeostasis, such as increasing mRNA levels of the farnesoid X receptor and the small heterodimer partner, decreasing mRNA levels of 3-hydroxy-3-Methylglutaryl-CoA reductase and up-regulating the mRNA expressions of ATP-binding cassette transporter-1 [154].

However, the biological effects of non-absorbable and highly polymeric procyanidins are not completely understood. Still, administration of these substances to high-fat/high-sucrose diet-induced mice attenuated the weight gain and inflammation, while metabolic urine profiling demonstrated that polymeric procyanidins administration also reduced the level of endogenous metabolites associated with insulin resistance. Furthermore, polymeric procyanidins treatment further decreased the Firmicutes/Bacteroidetes ratio and increased the abundance of *Akkermansia* [151].

Research has shown that resveratrol administration reduces the *Firmicutes* to *Bacteroidetes* ratio in mice fed a high-fat diet. Resveratrol treatment also inhibited the growth of *Enterococcus faecalis* and increased the growth of *Bifidobacterium* and *Lactobacillus*. Resveratrol administration further decreased adipogenesis, fatty acids synthesis and lipogenesis [155].

Recent metagenomic analysis has revealed that administration of green tea polyphenols to obese mice ameliorated obesity-induced dysbiosis. The authors of this study reported that green tea polyphenols brought about a significant reduction in the *Firmicutes/Bacteroidetes* ratio [156].

The results of a study by Ashley et al. [157] show that the consumption of sorghum polyphenols improves intestinal health by strengthening and stimulating *Lactobacillus*, *Bifidobacterium*, *Roseburia* and *Prevotella* species. It was also found that polyphenols from blueberries reduce obesity induced by a high-fat diet in mice by positively modulating the content and diversity of gut bacteria [146].

Fat storage and the resulting oxidative stress are the major pathogenic mechanisms of metabolic syndrome mediated by obesity in humans and mice [158]. Remely et al. [125] observed that the administration of (-)—epigallocatechin-3-gallate (EGCG) in high fat-fed mice had a significant effect on the gut microbiome in that the *Firmicutes/Bacteroidetes* ratio was significantly lower in mice supplemented with EGCG. Remely et al. [125] concluded that the beneficial effects of EGCG may be largely due to its antioxidant properties.

Another study revealed that polyphenols reduce microbiota dysbiosis-mediated metabolic syndrome by scavenging intestinal reactive oxygen species. In this work, polyphenolic compounds from grape increased the beneficial anaerobic gut bacteria, such as *Akkermansia muciniphila*, which was reported to be associated with improved metabolic status in humans [151]. Research conducted by Li et al. [159] indicates that sinapine administration suppresses non-alcoholic fatty liver disease by altering the intestinal microbiota in high-fat diet-induced mice. In this study, sinapine decreased the *Firmicutes* to *Bacteroidetes* ratio and increased the abundance of *Lactobacillaceae*, *Akkermansiaceae* and *Blautia*. Furthermore, supplementation of green tea polyphenol to female mice decreases the fasting blood glucose levels and mesenteric fat and increases the serum insulin levels via protecting against β-cell damage [160]. Finally, a control trial study reported that consumption of polyphenol-rich orange juice decreases the level of blood glucose and lipids via positively altering the gut microbiota by increasing the population of *Bifidobacterium* spp. and *Lactobacillus* spp. [145]. A controlled clinical study with a temporal series intergroup design evaluated 10 apparently healthy women (28.5  ±  8.4 years, 24.1  ±  3.3 kg/m^2^) after continuous consumption of commercial pasteurized orange juice for 2 months. After 60 days of orange juice consumption, there was a significant reduction in blood glucose by 6.3%, insulin by 33%, triglycerides by 30%, total cholesterol by 14% and LDL-C by 16% [145].

The above evidence suggests that polyphenolic compounds could suppress metabolic diseases via positively modulating the gut microbiome and increasing the growth of beneficial bacteria, with a concomitant decrease in the growth of harmful bacteria.

Some polyphenols and products of their metabolism have a negative effect on human organisms. Quercetin and rutin can be transformed by the GM to 3,4-dihydrophenylacetic acid (DOPAC), which is a metabolite of the neurotransmitter dopamine. DOPAC has anticancer, anti-inflammatory, cardioprotective and neuroprotective properties. However, in the presence of the NO radical, DOPAC inhibits mitochondrial respiration in isolated brain mitochondria, leading to mitochondrial dysfunction, which might be an important mechanism involved in the neurodegeneration associated with Parkinson’s disease [161]. Moreover, flavonoids can change the activity of phase I and II metabolizing enzymes (e.g., cytochrome P450, P-form phenol sulfotransferase, glutathione S-transferase and UDP-glucuronyl transferase).

Excessive intake of many polyphenolic compounds seems to exert an adverse effect on the body [162]. In the presence of the GM, O_2_ and transition metals, some polyphenols may act as pro-oxidants, leading to damage of DNA, lipids and other biological molecules. This pro-oxidant activity can be applied to cancer therapy and has a beneficial impact on the host cell, but if uncontrolled, the effect will be detrimental [5].

## 9. Conclusions and Future Perspective

The gut microbiota plays a central role in many mechanisms crucial for host physiology and metabolism. Several factors including age, unhealthy dietary habits, lack of exercise, stress, drugs and xenobiotics are responsible for dysbiosis, which has been associated with metabolic and gastrointestinal diseases. Polyphenols, present in a wide range of healthy foods, have been linked with beneficial action on multiple disorders, including obesity, diabetes and cardiometabolic and neurodegenerative diseases, which might be due to their antioxidant and anti-inflammatory effects. Evidence suggests that polyphenols are able to express prebiotic properties and exert antimicrobial activities against pathogenic gut microflora. Dietary polyphenols have shown benefits in distinct disorders, accompanied by a major impact on the GM towards symbiosis. Unfortunately, the therapeutic effects of polyphenols have been seriously compromised by the low bioavailability. Hence, there are some crucial challenges such as improving insufficient bioavailability, as in in vitro and in vivo studies, this situation makes it difficult to achieve advantageous results.

In order to improve the bioavailability of the extracted polyphenols, it would be advantageous to use appropriate technological processes, for example, micro- and nanoencapsulation or fermentation. Another significant challenge is to determine the appropriate dosage level of the food design, taking into account the changes in polyphenol content during production. The polyphenol interaction with the gut microbiota influences how many doses are needed to achieve the optimal therapeutic effects. Furthermore, the usage of polyphenolic compounds as prebiotics is also a substantial challenge, as their role as prebiotics in the human gut varies, depending on the residing probiotic strain. It is thus justified to conduct further research with a larger number of patients in order to better understand the issue and the hypothetical application of these solutions in therapy.

## Figures and Tables

**Table 1:** Effects of polyphenols on gut microbiota modulation.

Polyphenol	Dose and Time of Use (for Ani-Mal and Human Study)	Type of Study and Name of the Species	Changes in Microbiota	Ref.
Quercetin	4, 20, 50 µg/mL in medium	In vitro study	↓ *Ruminococcus gauvreauii*, *Bacteroides galacturonicus*, *Lactobacillus* sp.	[120]
Kaempferol, quercetin, myricetin and fisetin	25 μM in medium	In vitro study	Little or no antibacterial effect against *Bifidobacterium adolescentis*	[113]
Tannic acid	100.5 mg/mL in medium	In vitro study	↑ *Lactobacillus acidophilus*	[121]
(c)-epicatechin	150 mg/L and 1000 mg/L	In vitro study	↑ *Clostridium coccoides–Eubacterium rectale* group, *Bifidobacterium* spp. and *Escherichia coli* ↓ *C. histolyticum*	[122]
(+)-catechin	150 mg/L and 1000 mg/L	In vitro study	↑ *C. coccoides–Eubacterium rectale* group	[122]
Curcumin	100 mg/kg/day for 15 days	Animal study (mouse)	↑ Prevotellaceae, Bacteroidaceae ↓ Rikenellaceae	[123]
Picetannol	0.25% in diet for 18 weeks	Animal study (mouse)	↑ *Firmicutes*, *Lactobacillus* ↓ *Bacteroidetes*	[124]
(−)-Epigallocatechin-3-gallate	25 mg/kg/day for 4 months	Animal study (mouse)	↓ *Firmicutes/Bacteroidetes* ratio	[125]
Polymeric procyanidins	0.5% in diet for 20 weeks	Animal study (mouse)	↑*Akkermansia* ↓ *Clostridium*, *Lachnospiraceae*, *Bifidobacterium* ↓ *Firmicutes/Bacteroidetes* ratio	[126]
Daidzein	20 mg/kg/day during adulthood	Animal study (mouse)	Not specified	[127]
Picetannol (resveratrol analogue)	45 mg/kg/day for 6 weeks	Animal study (rat)	Nonsignificant changes in *Bacteroides* and *Firmicutes*	[128]
Quercetin	30 mg/kg/day for 6 weeks	Animal study (rat)	↓ *Erysipelotrichaceae*, *Bacillus*, *Eubacterium cylindroides*	[129]
Polyphenon G^®^ powder (purified preparation of tea-derived catechins)	0.2% Polyphenon G^®^ (0.07% tea catechins) for 3 weeks	Human intervention	↑ *Lactobacilli* ↓ *Enterobacteriaceae*	[130]
Isoflavones	100 mg/day for 15 days	Human intervention (postmenopausal women)	↑ stimulated dominant microorganisms of the *Clostridium coccoides-Eubacterium rectale cluster*, *Lactobacillus-Enterococcus* group, *Faecalibacterium prausnitzii* subgroup and *Bifidobacterium* genus	[131]

## Data Availability

Not applicable.
